# Non-standard amino acid incorporation into proteins using *Escherichia coli* cell-free protein synthesis

**DOI:** 10.3389/fchem.2014.00034

**Published:** 2014-06-10

**Authors:** Seok Hoon Hong, Yong-Chan Kwon, Michael C. Jewett

**Affiliations:** ^1^Chemical and Biological Engineering, Northwestern UniversityEvanston, IL, USA; ^2^Chemistry of Life Processes Institute, Northwestern UniversityEvanston, IL, USA; ^3^Robert H. Lurie Comprehensive Cancer Center, Northwestern UniversityChicago, IL, USA; ^4^Institute of Bionanotechnology in Medicine, Northwestern UniversityChicago, IL, USA

**Keywords:** non-standard amino acids, cell-free protein synthesis, synthetic biology, sequence-defined polymers, genome engineering

## Abstract

Incorporating non-standard amino acids (NSAAs) into proteins enables new chemical properties, new structures, and new functions. In recent years, improvements in cell-free protein synthesis (CFPS) systems have opened the way to accurate and efficient incorporation of NSAAs into proteins. The driving force behind this development has been three-fold. First, a technical renaissance has enabled high-yielding (>1 g/L) and long-lasting (>10 h in batch operation) CFPS in systems derived from *Escherichia coli*. Second, the efficiency of orthogonal translation systems (OTSs) has improved. Third, the open nature of the CFPS platform has brought about an unprecedented level of control and freedom of design. Here, we review recent developments in CFPS platforms designed to precisely incorporate NSAAs. In the coming years, we anticipate that CFPS systems will impact efforts to elucidate structure/function relationships of proteins and to make biomaterials and sequence-defined biopolymers for medical and industrial applications.

## Introduction

The incorporation of non-standard amino acids (NSAAs) into proteins and (poly)peptide-based materials is a key emerging application area in synthetic biology (Liu and Schultz, 2010; Hoesl and Budisa, 2012). In recent years, efforts to incorporate NSAAs using cell-free protein synthesis (CFPS) systems based on *Escherichia coli* have grown significantly. In this mini-review, we discuss these efforts, beginning with a description of the molecular basis for NSAA incorporation in *E. coli* using orthogonal translation systems (OTSs). We then describe CFPS and recent improvements in NSAA incorporation in crude cell extract as well as reconstituted systems of purified components. Finally, we discuss emerging frontiers and opportunities for CFPS.

## NSAA incorporation

To date, over 100 OTSs have been established for site-specific incorporation of NSAAs into proteins (O'Donoghue et al., 2013). Site-specific NSAA incorporation has been used to expand our understanding of biological systems by enabling studies of protein structure and dynamics with unique IR and X-ray diffraction signatures, fluorescent probes, and photocages (Liu and Schultz, 2010). In other examples, cross-linkable NSAAs have been incorporated to characterize protein-protein and protein-nucleic acid interactions (Liu and Schultz, 2010). In addition to expanding the chemistry of biomolecular systems, NSAA technology has also enabled researchers to mimic post-translational modifications of eukaryotic proteins in bacterial protein expression systems. In an exemplary model, site-specific acetylation of recombinant histones by genetically encoding acetyl-lysine (AcK) elucidated new mechanistic understanding (Neumann et al., 2009).

Beyond fundamental science, NSAA incorporation has also opened the way to novel biopolymer materials, enzymes, and therapeutics which are difficult—if not impossible—to create by other means. Antibody drug conjugates (Zimmerman et al., 2014), modified human therapeutics (Cho et al., 2011), tethered enzymes (Smith et al., 2013), protein polymers (Albayrak and Swartz, in press), phosphoproteins (Park et al., 2011), and selenoproteins (Bröcker et al., 2014) showcase the power of NSAA incorporation. In one example, pegylated human growth hormone showed improved potency and reduced injection frequency (Cho et al., 2011). In another case, an Anti-Her2 antibody bearing *p*-acetyl-L-phenylalanine enabled precise control of conjugation site and stoichiometry for selective and efficient conjugation to an anti-cancer drug resulting in enhanced tumor regression (Axup et al., 2012). These and other recent breakthroughs highlight exciting opportunities for expanding the chemistry of life.

To incorporate NSAAs site-specifically into proteins, OTSs require (re-)assignment of codons to NSAAs, NSAA-transfer RNA (tRNA) substrates, and ribosome selection of these non-natural substrates into the catalytic center. So far, ribosome accommodation of NSAAs has not been the limiting factor. Rather, strategies to provide for efficient and accurate incorporation of NSAA-tRNA substrates have been the biggest challenge. In practice, this is usually achieved by using orthogonal tRNA (o-tRNA)/aminoacyl-tRNA synthetase (o-aaRS) pairs from phylogenetically distant organisms (Kim et al., 2013). For example, an engineered tRNA^Tyr^_CUA_/TyrRS pair derived from *Methanocaldococcus jannaschii* is used frequently for NSAA incorporation (Wang et al., 2001). More recent expansions of the technology have used variants of the pyrrolysine translation system, tRNA^Pyl^_CUA_/PylRS from *Methanosarcinaceae* species (Polycarpo et al., 2006; Wang et al., 2012c). There are many seminal works of orthogonal pairs that have been developed for NSAA incorporation to help drive the field forward (Hughes and Ellington, 2010; Wan et al., 2010; Young et al., 2011; Bianco et al., 2012; Wang et al., 2012a,b; Ko et al., 2013; Lee et al., 2013; Niu et al., 2013; Bröcker et al., 2014; Ma et al., 2014). For codon selection, researchers tend to incorporate NSAAs in response to a non-sense stop codon or quadruplet codon (Wang et al., 2007; Neumann et al., 2010; Niu et al., 2013). The amber codon (TAG) has been the most widely used, because of its low frequency as a stop signal compared to other stop codons (TAA, TGA) (Hoesl and Budisa, 2012).

**Figure 1:**
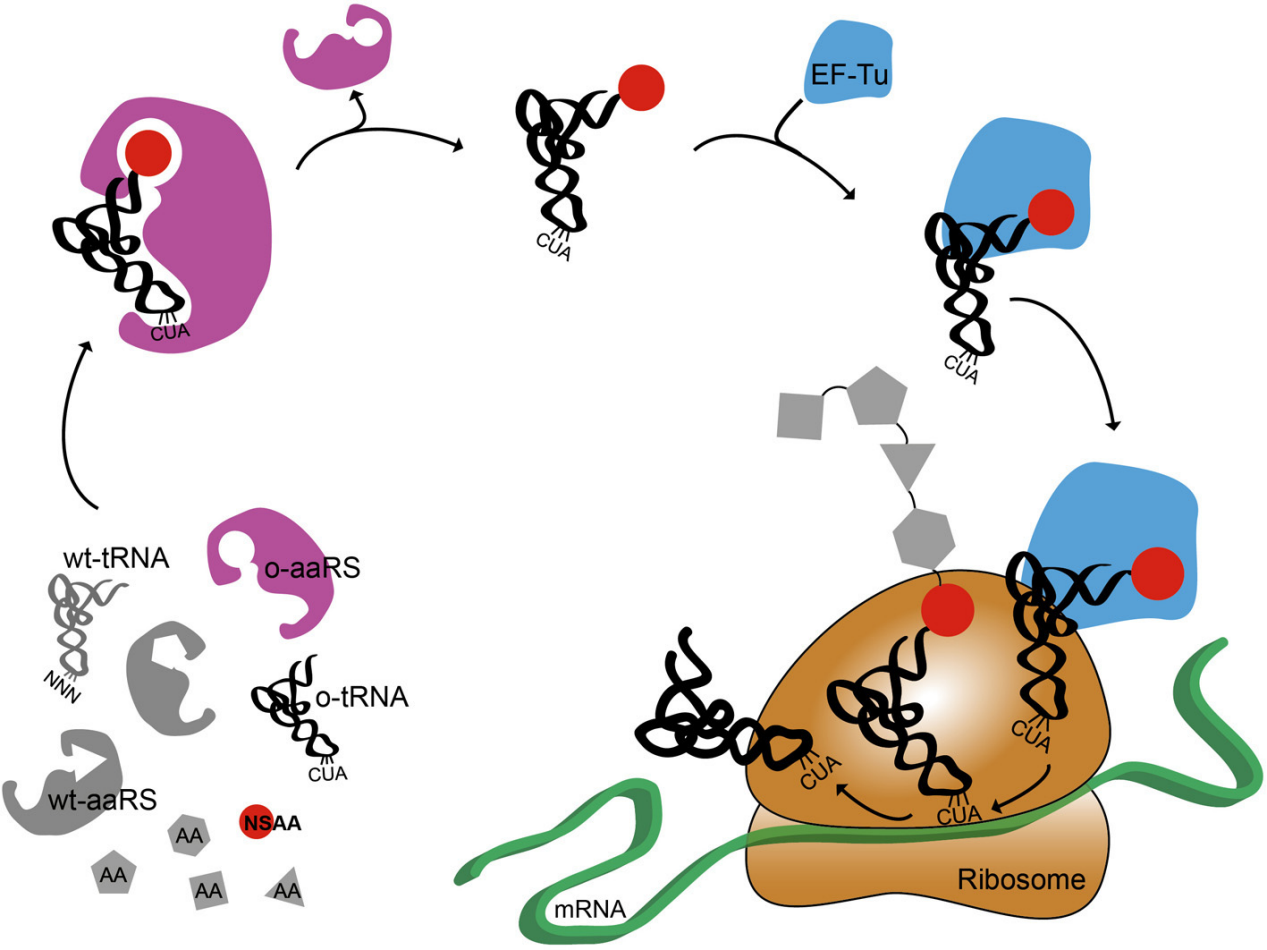
**Schematic representation of non-standard amino acid incorporation using an orthogonal translation system**. Orthogonal aminoacyl tRNA synthetase, o-aaRS; orthogonal tRNA, o-tRNA; wild-type aminoacyl tRNA synthetase, wt-aaRS; wild-type tRNA, wt-tRNA; elongation factor Tu, EF-Tu,; non-standard amino acid, NSAA. Anti-codon sequence on wt-tRNA is NNN, where N is A, C, G, or U. Anti-amber codon sequence on o-tRNA is CUA.

Figure 1 shows a cartoon representation of an OTS for amber suppression. It also highlights the systems biology challenges associated with NSAA incorporation (O'Donoghue et al., 2013). The orthogonal synthetases have poor catalytic efficiency (Tanrikulu et al., 2009; Nehring et al., 2012; Umehara et al., 2012). Elongation Factor Tu (EF-Tu) has a limited capability to incorporate bulky or charged NSAAs (Park et al., 2011; O'Donoghue et al., 2013). The presence of release factor 1 (RF1) can cause early termination of proteins when using amber suppression technology (Johnson et al., 2011; Hong et al., 2014). Recent advances have addressed some of these challenges by improving NSAA incorporation efficiency by engineering o-tRNA (Young et al., 2010; Chatterjee et al., 2012), o-aaRS (Liu et al., 1997; Chatterjee et al., 2012), or EF-Tu (Doi et al., 2007; Park et al., 2011) as well as controlling transcription and translation rate (Young et al., 2010; Chatterjee et al., 2013), and removing RF1 competition (Mukai et al., 2010; Johnson et al., 2011; Loscha et al., 2012; Lajoie et al., 2013). While further efforts to re-engineer translation are still needed, these improvements are accelerating rapid growth in synthetic biology efforts to “upgrade protein synthesis” (O'Donoghue et al., 2013). The bulk of this work is being carried out *in vivo;* however, complementary *in vitro* systems are also emerging, which we focus on below.

## Cell-free protein synthesis

**Figure 2:**
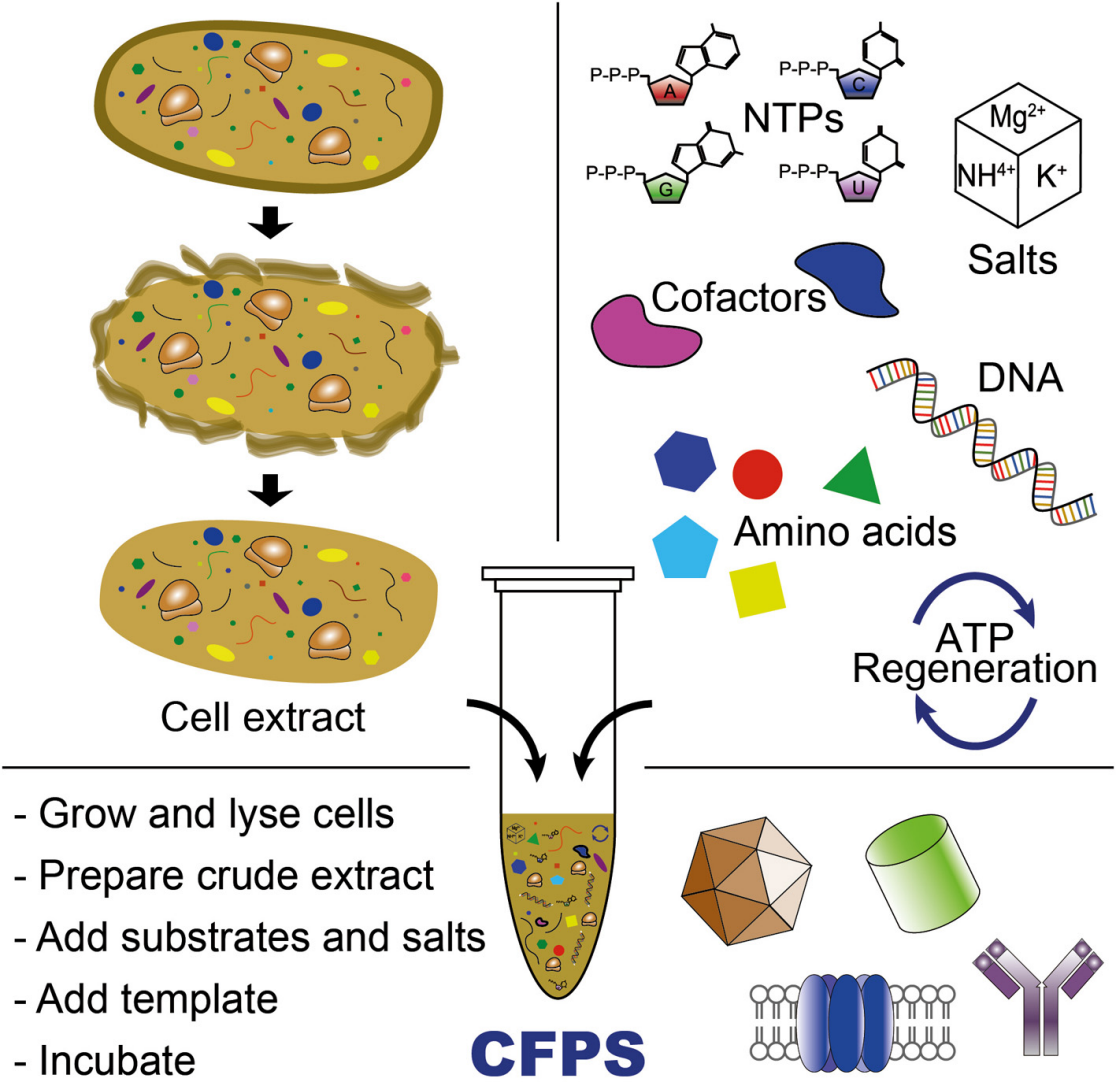
**Cell-free protein synthesis system for producing proteins or (poly)peptide-based materials**. CFPS requires cell extract, an energy regeneration system, and chemical substrates and salts (e.g., NTPs, amino acids, salts, and cofactors). Cell-free transcription and translation is initiated by adding DNA template (plasmid or PCR-amplified linear DNA templates) into the CFPS reaction.

CFPS is the synthesis of proteins *in vitro* without using intact, living cells (Jewett et al., 2008; Caschera and Noireaux, 2014). Over the last 50 years, CFPS systems have significantly advanced our ability to understand, exploit, and expand the capabilities of biological systems (Carlson et al., 2012; Swartz, 2012; Murray and Baliga, 2013). As a complement to *in vivo* systems, CFPS systems offer some interesting benefits. First, the open environment of the reaction allows the user to directly influence the biochemical systems of interest and as a result, new components can be added or synthesized and can be maintained at precise concentrations (Figure 2). For example, NSAAs that do not enter the cell can be utilized in CFPS. Second, cell-free systems are not constrained by cell-viability requirements, allowing protein synthesis to proceed with otherwise toxic reagents or protein products. Third, CFPS systems can use linear DNA fragments (e.g., PCR products) for a target gene expression, which avoids time-consuming gene cloning steps commonly required for *in vivo* protein synthesis. Finally, from a biomanufacturing perspective, cell-free systems separate catalyst synthesis (cell growth) from catalyst utilization (protein production) (Swartz, 2012). This concept represents a significant departure from cell-based processes that rely on microscopic cellular “reactors.”

Although CFPS technologies offer many exciting advantages, challenges remain that provide opportunity for improvement. For example, CFPS platforms still have few examples industrially. In addition, cell lysis procedures can be difficult to standardize, leading to different extract performance and limited reaction scales for academic research labs. Thus, while protein yields (mg/L) are often higher in CFPS, the total amount of protein purified from cells in research labs is typically more because the reaction scales are greater. Despite these challenges, the advantages of CFPS are stimulating new application areas. Dominant amongst these are high-throughput protein production (Calhoun and Swartz, 2005; Swartz, 2012; Catherine et al., 2013; Chappell et al., 2013; Murray and Baliga, 2013), clinical manufacture of protein therapeutics (Murray and Baliga, 2013), genetic circuit optimization (Shin and Noireaux, 2012), the construction of synthetic ribosomes (Jewett et al., 2013), and incorporation of NSAAs (Goerke and Swartz, 2009; Bundy and Swartz, 2010; Ugwumba et al., 2010; Mukai et al., 2011; Ugwumba et al., 2011; Loscha et al., 2012; Albayrak and Swartz, 2013a; Hong et al., 2014; Shrestha et al., 2014).

## Crude extract-based CFPS for NSAA incorporation

Efforts to use crude extract-based CFPS for the production of proteins containing single and multiple NSAAs are rapidly increasing. Key advances have centered on optimizing the performance of OTSs, expressing the OTS components in the source strain to create one-pot reactions, and removing RF1 competition.

### OTS optimization

The Swartz group has made marked contributions to CFPS development for high yielding NSAA incorporation (Goerke and Swartz, 2009; Bundy and Swartz, 2010). Showcasing the freedom of design in adjusting cell-free system components by direct addition to the reaction, their approach typically adds the NSAA and its purified o-aaRS directly to the reaction, while the o-tRNA is expressed during the cell growth prior to making the extract. As compared to *in vivo* systems, an advantage of this approach is that the toxicity associated with overexpressing the o-tRNA and o-aaRS is not observed. This is because the OTS elements are sequestered from each other until the protein synthesis reaction. Another advantage is that NSAAs with low solubility or poor transport characteristics can be used. For example, the tyrosine analog *p*-propargyloxy-L-phenylalanine (pPaF), which can be used in site-specific bioconjugation with the copper-catalyzed azide-alkyne cycloaddition, has low solubility. This is a known limitation *in vivo.* However, site-specific pPaF incorporation in the CFPS reaction was improved ~27-fold (as based on protein yield) for producing a modified protein when compared to previous *in vivo* approach (Bundy and Swartz, 2010).

Cell-free systems are not only useful for making protein product but also for assessing the catalytic efficiency of the OTSs. A growing number of studies, for example, have shown that o-aaRSs are poor catalysts, up to 1000 times worse than natural aminoacyl tRNA synthetases, mainly due to the fact that the evolution of the orthogonal pairs occurs under high concentrations of non-standard amino acids (Tanrikulu et al., 2009; Nehring et al., 2012; Umehara et al., 2012; Albayrak and Swartz, 2013b). Future efforts for improving site-specific NSAA incorporation will require the development of o-aaRSs with higher catalytic rates and stronger affinity for the o-tRNAs. One approach to achieve such desired properties is to find strategies to remove fitness and the health of the cell on evolutionary outcomes. Ellington's lab recently published such an approach, compartmentalized partnered replication (Ellefson et al., 2014), but there are other opportunities as well.

In the meantime, NSAA incorporation in cell-free systems is being improved by increasing the amount of o-tRNA and o-aaRS in the CFPS reaction. One approach to achieving increased o-tRNA levels was pioneered by Albayrak and Swartz (2013a) and validated by Hong et al. (2014). Namely, the o-tRNA is co-produced in the CFPS reaction as a transzyme construct. The transzyme construct is a DNA fragment containing hammer-head ribozyme sequence between T7-controlled promoter and o-tRNA sequences. Upon transcription, the hammer-head ribozyme cleaves 5′-end of tRNA liberating active tRNA into the reaction (Fechter et al., 1998) and thereby increased o-tRNA is supplied to the CFPS reaction. With the transzyme technology, up to 0.9–1.7 mg/mL of a modified protein containing NSAA was produced (Albayrak and Swartz, 2013a) and multiple site NSAA incorporation was improved (Hong et al., 2014). As another approach, there are efforts to co-express all the OTS components in the source strain. While there are potential concerns of expressing both the o-tRNA and the o-aaRS in the source strain prior to lysis, Bundy and colleagues recently showed that this was not only possible, but improved CFPS yields of a modified protein (Smith et al., 2014). As an alternative approach, natural amino acids have been depleted from crude extracts to allow for the incorporation of NSAA analogs (Singh-Blom et al., 2014).

### Removing RF1 competition

NSAA incorporation using amber codon suppression is limited by RF1 competition (Lajoie et al., 2013). The presence of RF1 causes the production of truncated protein and low yields of protein product in the case of multiple identical site-specific NSAA incorporation (Park et al., 2011; Hong et al., 2014). Deletion of RF1 is lethal in native biological systems. However, this limitation was recently addressed by making a more promiscuous release factor 2 (Johnson et al., 2011, 2012), and genome engineering (Mukai et al., 2010; Heinemann et al., 2012; Ohtake et al., 2012). Most notably, the development of the first genomically recoded *E. coli* strain was completed; all 321 TAG stop codons were reassigned to synonymous TAA codons allowing the deletion of RF1 without observing growth defects (Lajoie et al., 2013).

With RF1-deficient *E. coli* strains at hand, efforts are underway to utilize these strains *in vivo* for improved production of proteins with NSAAs, but also to develop RF1-deficient CFPS systems. In one example, human histone H4 protein was produced with site-specific incorporation of AcK at four amber sites by using a RF1-deficient cell extract (Mukai et al., 2011). In another case, the effect of RF1 deletion was systematically assessed for single and multiple site pPaF incorporation using cell extracts from genomically recoded *E. coli* with or without RF1 (Hong et al., 2014). The production of modified soluble superfolder green fluorescent protein (sfGFP) containing pPaF was 2.5-fold higher in the RF1-deficient cell extract compared to the RF1-present cell extract. The authors showed that the yield improvement was due to an increase in full-length modified sfGFP synthesis, observing a shift from 20% full-length product (with RF1) to 80% full-length product (without RF1). In a complementary approach, RF1-depleted cell extracts were constructed from selective removal of a RF1 variant tagged with chitin-binding domains (Loscha et al., 2012) or His-tag (Gerrits et al., 2007). Looking forward, we anticipate that RF1-deficient *E. coli* strains will become an important chassis for NSAA incorporation.

## Reconstituted *in vitro* translation for NSAA incorporation

Although crude extract-based CFPS systems have shown tremendous growth, there are limitations to the number of open coding channels available because one must grow *E. coli* to obtain cellular lysate. To address this limitation, researchers have turned to purified translation systems, such as the PURE system (protein synthesis using purified recombinant elements) (Shimizu et al., 2001). Since the user defines all of the elements in the PURE system, single or multiple components (e.g., tRNA, aaRS) can be omitted, increased, or decreased according to the experimental purpose (Hirao et al., 2009). This enables highly efficient sense and non-sense suppression and provides unmatched flexibility for genetic code reprogramming to incorporate NSAAs (Shimizu et al., 2005). Efforts using purified translation for NSAA incorporation have mainly centered on the production, screening, and selection of peptidomimetic, or non-standard peptides (Josephson et al., 2005; Tan et al., 2005; Hartman et al., 2007; Passioura and Suga, 2013). As an exemplary illustration, peptidomimetic synthesis was achieved by adding pre-aminoacylated tRNA with NSAAs corresponding to sense codons in the reconstituted translation system lacking aaRS activities (Forster et al., 2003). In an alternative approach, Suga's group has leveraged the highly flexible tRNA acylation Flexizyme technology. Flexizyme is an artificial ribozyme that was developed to charge virtually any amino acid onto any tRNA *in vitro*, allowing the synthesis of proteins and short peptides containing multiple distinct NSAAs (Murakami et al., 2006; Ohuchi et al., 2007). A drug discovery pipeline has been enabled by combining a modified reconstituted translation system with Flexizyme technology (Goto et al., 2011) for the development of small peptides (Passioura and Suga, 2013), such as macrocyclic peptides (Hayashi et al., 2012; Morimoto et al., 2012). In yet a different approach, Szostak's work has demonstrated the ability to incorporate numerous amino acid analogs using the endogenous machinery. Strikingly, the natural aaRS machinery tolerates many kinds of side chain derivatives, such as α,α disubstituted, *N*-methyl and α-hydroxy derivatives (Hartman et al., 2007). Even D-amino acids have been shown to be compatible with polypeptide elongation (Fujino et al., 2013).

Although PURE translation is a powerful research tool, the cost of the PURE system is prohibitive for most commercial applications. For example, when compared to crude extract-based CFPS systems, which have been scaled to 100 L (Zawada et al., 2011), the PURE system costs ~1000 times more on a milligram protein produced/$ basis (Hong et al., 2014) and yields lower protein titers than the crude extract-based CFPS system (Lee et al., 2012; Hong et al., 2014). Hence, an important design decision for producing proteins with NSAAs using cell-free systems is choosing between a crude extract and a purified system.

## Emerging applications

Marked advancements in productivity, improvements in OTS efficiency, and increases in the ability to incorporate multiple identical NSAAs (in crude extracts) and multiple distinct NSAAs (in the PURE system) are rapidly expanding the possible applications of CFPS systems. In this section, we highlight several emerging applications made possible by these advances. These include the production of protein-based materials and therapeutics.

### Protein-based materials

NSAA incorporation is being applied to create new types of sequence-defined polymers for versatile applications in biomaterials synthesis. In an illustrative example, Albayrak and Swartz reported direct polymerization of proteins containing two or three copies of site-specifically incorporated NSAAs that allows copper-catalyzed azide-alkyne cycloaddition to form linear or branched protein polymers (Albayrak and Swartz, in press).

### Therapeutics

NSAA incorporation is being applied to (i) clinical scale production of protein therapeutics and vaccines, (ii) discovery of novel biologics through ribosome display methods (Murray and Baliga, 2013), and (iii) structure/function studies to identify protein inhibitors. Swartz and colleagues, for example, have developed a novel pipeline for the production of decorated virus-like particles that could function as potential vaccines and imaging agents (Lu et al., 2013). In another example, Sutro Biopharma has demonstrated the synthesis of site-specific antibody drug conjugates (ADCs) (Zimmerman et al., 2014). Their ADCs, which were synthesized at ~250 μg/mL titers, proved potent in cell cytotoxicity assays. Rather than producing a therapeutic using CFPS, Ugwumba et al. utilized the NSAA 7-(hydroxy-coumarin-4-yl) ethylglycine to structurally probe a protein from the West Nile Virus to identify novel inhibitors (Ugwumba et al., 2011). Collectively, these recent reports highlight the utility of CFPS for producing novel vaccines and therapeutics, as well for serving as a rapid and attractive tool in drug discovery.

## Conclusion and outlook

CFPS has emerged as a promising approach to enable site-specific incorporation of NSAAs into proteins and bio-based polymers. With the ability to select peptides and proteins for novel drugs in the PURE system and advent of scalable CFPS from crude extract systems, we anticipate significant growth in the field in years to come. Immediate challenges are (i) the evolution of more efficient OTSs (ii) new codons that can be assigned to NSAAs, and (iii) the development of genomically recoded organisms for preparing highly active cellular extracts. Addressing these challenges and continuing to lower costs will expand the scale and scope of cell-free biology, providing a transformative toolbox that enables new frontiers in synthetic biology.

### Conflict of interest statement

The authors declare that the research was conducted in the absence of any commercial or financial relationships that could be construed as a potential conflict of interest.
